# When Does Magnesium Stearate Improve Carrier-Based Dry Powder Inhalers (DPI) Performance? A Critical Review of Material, Process, and Formulation Factors

**DOI:** 10.3390/ph19101535

**Published:** 2026-09-28

**Authors:** Aleksandra Rzewińska-Dąbrowska, Katarzyna Wójcik, Ewelina Juszczyk, Ewa Tratkiewicz, Przemysław Dorożyński

**Affiliations:** 1 Department of Drug Technology and Pharmaceutical Biotechnology, Faculty of Pharmacy, Medical University of Warsaw, Banacha 1, 02-097 Warszawa, Poland; 2 R&D Center, LEK-AM., Ostrzykowizna 14A, 05-170 Zakroczym, Poland; 3 R&D Center, Celon Pharma S.A., Marymoncka 15, 05-052 Kazuń Nowy, Poland; 4 Department of Inorganic and Analytical Chemistry, Faculty of Pharmacy, Jagiellonian University Medical College, Medyczna 9, 30-688 Kraków, Poland

**Keywords:** magnesium stearate, force control agent, carrier-based DPI, fine particle dose

## Abstract

Magnesium stearate (MgSt), although well established as a pharmaceutical lubricant in solid oral dosage forms, plays a more complex role in carrier-based dry powder inhalers (DPIs), where it functions as a surface modifier and force control agent (FCA). In DPI formulations, MgSt can influence powder rheology, moisture resistance, interparticulate interactions, and aerosolization efficiency; however, these effects depend not simply on its presence or concentration, but on whether effective surface modification is achieved. This review critically evaluates experimental evidence on MgSt-mediated particle coating in carrier-based DPIs, with particular emphasis on how material attributes, particle cohesion and surface area, process energy, coating strategy, and MgSt concentration interact to determine coating efficiency and formulation performance. By integrating these factors, the review aims to provide a mechanistic framework for selecting and optimizing MgSt coating strategies and to identify methodological priorities for future research. The available evidence indicates that process energy is a major determinant of coating efficiency. High-energy techniques, particularly mechanofusion, can effectively modify highly cohesive micronized particles that are difficult to coat using conventional blending, whereas high-shear blending may provide sufficient energy for surface modification of larger, coarse lactose carriers. Mechanical milling represents a distinct strategy in which particle-size reduction and surface modification occur simultaneously. Direct comparison between coating approaches remains difficult because studies differ in MgSt grade, carrier and API properties, particle size distribution, processing conditions, and methods used to assess coating efficiency. MgSt generally improves aerosolization by modifying cohesive and adhesive interactions, but the magnitude of this effect is formulation-specific. Improvements in fine-particle delivery depend on MgSt particle size and specific surface area, the properties and surface area of the particles being coated, process energy, and the resulting coating efficiency. Consequently, increasing MgSt concentration does not necessarily improve performance, and excessive amounts may lead to overlubrication or MgSt agglomeration. Despite considerable progress, important knowledge gaps remain regarding how MgSt coating translates into improved DPI performance. Further studies integrating material properties, processing conditions, surface modification, and formulation performance are needed to establish more robust relationships between coating strategy and functional outcomes. Such knowledge may support more rational and formulation-specific use of MgSt in carrier-based DPIs.

## 1. Introduction

The administration of an active substance directly to the lungs offers several advantages over oral delivery, including avoidance of the first-pass effect, rapid onset of action in the treatment of obstructive pulmonary diseases or acute asthma attacks, a relatively low required dose of the active ingredient, and a lower incidence of systemic side effects compared with other routes of administration [1,2]. However, the development of inhaled drugs, whether pressurized metered-dose inhalers (pMDI), dry-powder inhalers (DPI), soft-mist inhalers, or nebulizers, is a much greater challenge from a technological standpoint than the development of oral solid dosage forms. These dosage forms must simply be swallowed by a patient to reach the gastrointestinal tract, which is feasible for a large portion of the population. By contrast, an inhaled drug must be correctly aspirated by the patient through the inhaler, the formulation must be withdrawn from the device during inhalation, and an effective dose of the drug must leave the device without depositing on the inner walls of the inhaler and then reach the lower respiratory tract. Additionally, the active pharmaceutical ingredient (API) must be delivered to the proper region of the lungs, where the targeted receptors are located. For these reasons, manufacturing of inhaled drugs delivering efficient and reproducible doses remains challenging [3].

The two main types of inhalers used in the treatment of asthma and chronic obstructive pulmonary disease (COPD) are pMDIs and DPIs. However, the increasing emphasis on reducing carbon footprints has led many countries to restrict the use of pMDIs containing hydrofluorocarbons (HFCs), particularly HFC-134a and HFC-227ea, due to their significant greenhouse gas effects [4,5,6]. In contrast, DPIs do not require propellants, as the powder is dispersed by the patient’s inhalation maneuver. Moreover, DPIs are associated with higher patient adherence among individuals with asthma and COPD [7]. Therefore, when administration is feasible with both pMDI and DPI, the development of DPI-based platforms is a reasonable approach.

Drug particles must reach the lower airways to achieve a therapeutic effect. The main way to obtain particles within the respirable size range is the micronization process. However, this process can negatively affect the physicochemical properties of the particles by increasing specific surface area and the tendency of the particles to stick together (strong cohesive forces) or to surfaces (adhesive forces), which causes poor flowability and aerosolization [8,9,10]. To overcome this limitation, coarser carrier particles are used as an excipient [11,12]. This type of formulation was termed a carrier-based or adhesive mixture [13,14,15] and is still used today under the same name.

A carrier-based dry powder formulation contains APIs, micronized to a particle size below 5 µm in diameter, which are blended with a coarse lactose carrier and filled into a dry powder inhaler [16]. During blending, the API particles bind to the carrier surface through electrostatic interactions, capillary forces, and van der Waals interactions [17,18,19]. Due to significant discrepancies in size between particles, van der Waals interactions are sufficiently strong to maintain the bond [20]. Interaction between drug and carrier particles should provide expected blend homogeneity. Upon inhalation, the particles are carried by the airflow through the mouth and throat. The larger carrier particles tend to deposit at the back of the throat, while the API particles detach from the larger carrier surface and, together with the small amount of fine lactose particles, are delivered to the deep regions of the lungs [21]. Thus, although the API-carrier interaction must ensure a homogeneous mixture, it should also be weak enough to allow API detachment during inhalation. Managing this balance between interaction forces of API-carrier particles is already challenging. The addition of fine lactose particles or magnesium stearate, which is the topic of this review, adds another layer of complexity.

Carrier-based DPI formulations are designed to deliver locally acting small molecules. After the COVID-19 pandemic, carrier-free platforms, which can deliver larger molecules for a systemic effect, have gained more interest. Carrier-free platforms are fundamentally different from carrier-based ones and lie outside the scope of this review.

The number of approved excipients in commercially available carrier-based dry powder formulations remains limited. For many years, lactose monohydrate was the only excipient used in those types of products. Lactose is widely used and well established as a safe excipient, including in patients with lactose intolerance [12,22].

It was not until 2012 that formulations containing magnesium stearate (MgSt) in addition to lactose were introduced to the market [23]. MgSt is well established as a lubricant in conventional solid dosage forms, such as tablets and capsules [24,25]. However, its introduction into pulmonary drug delivery systems represented a significant development in the formulation of carrier-based DPIs.

Fourteen years after the introduction of MgSt into carrier-based DPIs, numerous studies have investigated its potential functions in these formulations [26,27] as well as particle-coating techniques [28] and methods for coating characterization. The current state of knowledge has also been comprehensively summarized in several recent review articles [21,29,30]. However, given the complexity of carrier-based DPI formulations, an important knowledge gap remains regarding how MgSt should be incorporated and processed within the formulation to achieve improved performance compared with formulations without MgSt.

The aim of this review is therefore to identify the material, formulation, and process factors that determine the effects of MgSt in carrier-based DPIs. Particular attention is given to the properties of the starting materials, manufacturing process, and sequence of ingredient addition. The review seeks to identify the conditions under which MgSt can improve product quality and performance, for example, by enhancing stability or increasing the fine particle dose (FPD).

## 2. Magnesium Stearate: Material Attributes Relevant to DPI Performance

Magnesium stearate (MgSt) is a hydrophobic surface-modifying excipient used in carrier-based dry powder inhalers to reduce interparticle adhesion, improve powder dispersion, and increase resistance to moisture. Its functionality depends strongly on material attributes such as source, fatty acid composition, crystallinity, hydration state, particle size distribution, morphology, and specific surface area. These properties determine the ability of MgSt particles to spread over lactose surfaces and form an effective coating layer.

MgSt may be obtained from vegetable or animal sources. Differences in raw-material origin can affect crystallinity, morphology, particle size, hydration behaviour, and fatty acid composition, leading to variability in coating efficiency and formulation performance. Vegetable-derived grades are increasingly preferred because of regulatory and safety considerations, although functionality remains grade- and supplier-dependent. The principal differences reported between vegetable- and bovine-derived MgSt are summarized in Table 1 [31].

Crystallinity, particle size, and specific surface area influence the ability of MgSt to delaminate and spread over carrier particles. A smaller particle size and larger available surface area may promote more uniform coating, whereas insufficient spreading can leave MgSt as separate agglomerates and limit its effect on aerosolization. The resulting hydrophobic layer reduces adhesive interactions and moisture uptake, but excessive or non-uniform coating may impair blend homogeneity or drug detachment. Therefore, MgSt source and grade should be selected on the basis of functional performance rather than nominal composition alone [32,33].

Fatty acid composition, particularly the relative content of stearate and palmitate, is another important material attribute. Grades with similar morphology, particle size, crystallinity, and specific surface area may nevertheless exhibit different coating and lubrication behaviour because of variations in their fatty acid profile [34]. For inhalation applications, specifications should therefore include fatty acid composition, particle size distribution, hydration state, and microbiological quality.

MgSt occurs in amorphous and crystalline forms and may exist as a monohydrate, dihydrate, or trihydrate [35]. The hydration state affects crystallinity, particle size, and interaction with atmospheric moisture. The monohydrate has been proposed as the most suitable form for carrier-based DPI formulations because of its relatively high crystallinity, smaller particle size, and improved stability under elevated humidity [36]. However, its performance remains dependent on the complete API–carrier–MgSt system and the coating process applied.

## 3. Overview of Commercial DPIs

Commercial carrier-based dry powder inhalers approved in the United States and Europe use either lactose alone or lactose combined with magnesium stearate (MgSt) [22,37,38,39]. Table 2 and Table 3 summarize representative products introduced between 1997 and 2020. Their composition shows that MgSt has not become a universal component of carrier-based DPI formulations. Products containing lactose as the sole carrier continued to receive marketing authorization after the introduction of MgSt-containing systems, indicating that its inclusion is formulation-specific rather than generally required.

The comparison of commercial products suggests that MgSt is used selectively, most likely to address formulation- and platform-specific challenges related to particle adhesion, moisture sensitivity, blend stability, or aerosolization. However, product composition alone cannot establish the functional necessity of MgSt. Commercial formulations differ not only in their active ingredient but also in lactose grade, particle-size distribution, manufacturing process, device resistance, dose, and intellectual-property constraints. Consequently, the presence or absence of MgSt should not be interpreted as direct evidence that a particular API intrinsically requires or does not require surface modification.

The glycopyrronium-containing products illustrate this limitation. Glycopyrronium is consistently formulated with MgSt in the listed Breezhaler products, whereas indacaterol is marketed both without MgSt as a single-agent product and with MgSt in combination formulations. This pattern suggests that MgSt may be particularly beneficial in glycopyrronium-containing systems, but it does not demonstrate that indacaterol maleate cannot be formulated without it. The observed association may also reflect common platform technology, carrier selection, manufacturing strategy, or product-specific performance requirements.

Similarly, the repeated use of MgSt in Ellipta products may indicate a platform-level formulation strategy rather than an API-specific requirement. The commercial evidence therefore supports a more cautious conclusion: MgSt is not a universal excipient for carrier-based DPIs, but a functional surface modifier employed when the combined API–carrier–process–device system requires adjustment of interparticulate interactions or protection against humidity.

Magnesium stearate can be laminated and smeared onto coarser particles in carrier-based formulations [40]. However, the effectiveness of magnesium stearate depends strongly on the powder coating process. If the coating process is inadequately selected or carried out under suboptimal parameters, effective coating may not be achieved. Given the importance of the coating process in the development of DPI formulations, the available coating techniques are discussed in detail in the following sections.

## 4. Dry Particle Coating Techniques

Several approaches have been proposed for modifying particle surfaces in dry powder inhalers (DPIs), including solvent-based (spray drying, fluidized-bed coating, solvent-assisted mixing), vapor-based (physical and chemical vapor deposition), and dry coating technologies [21,41,42]. Among these, dry coating has emerged as the most relevant strategy for carrier-based DPIs because it enables surface modification without exposing powders to solvents or subsequent drying steps, thereby preserving the physicochemical properties of both the carrier and the active pharmaceutical ingredient (API).

Unlike liquid-based coating methods, which are mainly applied to carrier-free inhalation systems, dry coating relies on mechanical energy to redistribute fine guest particles, such as magnesium stearate (MgSt), onto the surface of larger host particles. The effectiveness of this process depends not only on the coating technique itself but also on the balance between particle cohesion, adhesion, and the energy transferred during processing. Consequently, different coating methods are not interchangeable and should be selected according to the properties of the host particles and the intended function of the coating.

For coarse lactose carriers, moderate mechanical energy may be sufficient to achieve surface modification. In contrast, coating highly cohesive micronized particles requires substantially greater energy to overcome cohesive forces, disperse agglomerates, and expose individual particle surfaces. As a result, coating efficiency is governed by both material characteristics and process energy rather than by the presence of MgSt alone.

Depending on the applied energy and particle interactions, MgSt may form either discrete surface deposits or more continuous coating layers (Figure 1). Continuous coatings may consist of smooth films or porous monolayers of guest particles [17,20]. The extent and uniformity of surface coverage are expected to influence particle adhesion, moisture sensitivity, and aerosolization performance, although these relationships remain difficult to compare across studies because coating efficiency is rarely quantified using standardized analytical methods [43].

Dry coating should therefore be regarded not simply as a manufacturing step but as a particle-engineering strategy that modifies interfacial properties, including cohesion, adhesion, electrostatic interactions, and moisture sensitivity [20]. However, the different dry coating technologies generate markedly different stress conditions and consequently cannot be expected to produce equivalent coating structures or formulation performance.

### 4.1. Mechanofusion

Mechanofusion is the highest-energy dry coating technique applied in pharmaceutical powder engineering. The process subjects powder particles to repeated compression, shear, friction, and rolling forces within a rotating chamber, promoting both deagglomeration and intimate contact between host and guest particles (Figure 2) [21,43].

Unlike conventional blending, mechanofusion is capable of coating highly cohesive micronized particles because the applied mechanical energy exceeds the cohesive forces responsible for agglomeration. Consequently, it enables the formation of thin and relatively uniform MgSt layers without substantially changing the particle size distribution [45]. This characteristic makes mechanofusion particularly suitable for modifying fine APIs or cohesive lactose grades.

Nevertheless, the high energy input is accompanied by practical limitations. Local temperature increases may influence coating behaviour or powder stability, making process cooling and optimization of process parameters essential for reproducible coating quality. Furthermore, the greater complexity, energy consumption, and limited industrial implementation of mechanofusion may restrict its routine use compared with conventional blending.

### 4.2. Mixing

Blending remains the most widely used coating approach in commercial DPI manufacturing because it is readily scalable and compatible with existing pharmaceutical equipment [46,47,48,49,50].

However, its coating capability depends strongly on the energy input.

Low-shear blending primarily produces homogeneous mixtures with little surface modification, whereas high-shear blending generates sufficient mechanical stress to redistribute MgSt over the surface of coarse lactose carriers. Accordingly, the function of high-shear blending extends beyond mixing to partial surface engineering.

The distinction between low- and high-shear processing is particularly important because many publications refer to both as “mixing”, despite producing substantially different particle structures. Low-shear blending is generally ineffective for coating highly cohesive micronized particles, whereas high-shear blending can successfully modify larger carrier particles (typically 45–200 µm) [49]. This indicates that coating efficiency depends not only on mixer design but also on the relationship between particle size, cohesion, and the mechanical energy delivered to the powder bed.

Alternative dry blending technologies, including the Theta Composer, similarly rely on shear and compression forces to enhance host–guest interactions [20]. Although successful coating has been demonstrated using conventional blending techniques [51,52,53], the degree of surface coverage is generally lower than that achieved by mechanofusion, particularly for cohesive powders.

### 4.3. Mechanical Milling

Jet-milling is a particle-size reduction process that enables the micronization of crystalline materials. During jet-milling, high-velocity gas streams generate interparticle and particle-wall collisions, providing sufficient energy to reduce crystals to fine, respirable particles with relatively narrow particle size distributions [27]. The technique, like blending, is widely employed in the manufacture of carrier-based dry powder formulations intended for pulmonary drug delivery [1,26,27,54].

The particles produced by jet-milling may exhibit irregular shapes, rough surfaces, increased electrostatic charge, and strong interparticle cohesive forces. These characteristics can adversely affect powder handling, flowability, and aerosolization performance [27,55,56]. Consequently, the micronized API is commonly blended with a coarser carrier material in a subsequent step during the preparation of carrier-based dry powder formulations. The coarser carrier facilitates powder handling, dispersion, and aerosolization.

An alternative approach is the co-jet milling of the API with a lubricant [57]. This strategy enables particle size reduction and surface modification to occur simultaneously. Co-milling with MgSt can reduce interparticulate interactions by forming low surface energy coatings and thereby improve the fine particle fraction [57,58].

Overall, the available evidence suggests that process energy is one of the principal determinants of coating efficiency. Low-energy mixing is generally adequate for homogenization, moderate-energy high-shear blending effectively modifies coarse lactose carriers, whereas high-energy processes such as mechanofusion or co-milling are required to coat highly cohesive micronized particles. However, the lack of standardized methods for quantifying coating coverage and comparing energy input across different equipment remains a major limitation of the field. A comparative summary of the principal dry coating techniques is presented in Figure 3.

## 5. Selecting the Appropriate Coating Strategy for Carrier-Based DPIs

The effectiveness of MgSt coating is determined not by the coating technique itself, but by the relationship between process energy and the cohesive forces within the powder. Consequently, different coating methods should be regarded as complementary rather than competing technologies, each being suitable for a specific particle system. Mechanical milling, mechanofusion, and blending therefore address different formulation challenges.

Mechanical milling combines particle size reduction with simultaneous surface modification and has been proposed as a preprocessing step for APIs prior to preparation of the final carrier-based blend. This strategy has been described, for example, for glycopyrronium co-micronized with MgSt [40,59]. In contrast, blending is primarily intended for preparation of the final formulation, whereas mechanofusion provides sufficient energy to modify highly cohesive powders that cannot be effectively coated by conventional mixing.

### 5.1. The Challenge of Coating Micronized Particles

The principal limitation in coating micronized APIs is not the presence of MgSt but the high cohesive forces acting between particles. For micronized powders, these forces may exceed gravitational forces by up to fourfold, leading to stable agglomerates that prevent MgSt from accessing individual particle surfaces [60]. Consequently, successful coating requires an initial deagglomeration step before surface modification can occur.

This explains why conventional blending frequently fails to improve the performance of cohesive micronized powders. The mechanical energy generated during mixing is generally insufficient to disrupt agglomerates, leaving MgSt as separate particles rather than forming an effective surface coating.

Experimental evidence supports this interpretation. Zhou et al. compared mechanofusion and conventional blending for coating highly cohesive salbutamol sulphate (d90 = 7.8 µm), using X-ray Photoelectron Spectroscopy (XPS) to quantify MgSt coverage. Only mechanofusion produced measurable surface coating, whereas conventional blending resulted in no detectable differences from untreated particles [9]. Similar observations were reported for cohesive lactose (Pharmatose 450, d90 ≈ 45 µm), further demonstrating that coating efficiency depends primarily on overcoming particle cohesion rather than on the presence of MgSt itself [49].

All these studies indicate that for highly cohesive powders, process energy must first exceed the deagglomeration threshold before effective coating can occur.

### 5.2. Coating of Carrier Particles

The requirements are fundamentally different for coarse lactose carriers. Because carrier particles are substantially larger (typically up to 200 µm), cohesive forces are less dominant, and complete deagglomeration is no longer the limiting step. Instead, the objective becomes controlled modification of the carrier surface to optimize adhesive interactions with the micronized API.

Under these conditions, high-shear blending can achieve coating efficiencies comparable to mechanofusion. This was demonstrated using Inhalac 230 (d10 = 62 µm; d90 = 155 µm), where carrier particles were coated by either mechanofusion or high-shear blending before mixing with micronized ipratropium. Both approaches produced comparable improvements in aerodynamic performance relative to untreated carriers [46].

These findings suggest that increasing process energy beyond that required for effective carrier coating does not necessarily translate into further improvements in aerosolization. Instead, the optimal coating strategy depends on whether the formulation challenge is dominated by particle cohesion (micronized APIs) or by modulation of adhesive interactions between the API and the carrier.

### 5.3. Assessing Coating Efficiency

A major limitation in comparing coating strategies is the lack of standardized methods for quantifying surface coverage. Most studies infer coating efficiency indirectly from aerodynamic performance, despite the fact that similar fine particle fraction (FPF) values may arise from different coating mechanisms. Direct surface characterization using Time-of-Flight Secondary Ion Mass Spectrometry (ToF-SIMS) [61] or X-ray Photoelectron Spectroscopy (XPS) [61,62,63] provides considerably stronger evidence of successful coating but remains relatively uncommon.

Consequently, comparisons between coating technologies should be interpreted cautiously, as differences in reported performance may reflect variations in coating coverage, particle properties, or process conditions rather than intrinsic superiority of one technique over another.

## 6. Why Is Magnesium Stearate Used in Carrier-Based DPIs?

Commercial DPI products (Table 2 and Table 3) indicate that MgSt is not incorporated as a universal excipient but rather as a functional surface modifier introduced to overcome specific formulation challenges. Depending on the formulation, MgSt may reduce undesirable interparticulate interactions, improve aerosolization, or enhance resistance to humidity during storage. These functions are interrelated and originate from the ability of MgSt to modify particle surface properties rather than from its presence alone (Figure 4).

### 6.1. Modifying Interparticulate Interactions

The primary consequence of MgSt coating is the alteration of particle–particle interactions. Whether this translates into improved formulation performance depends on the extent of surface coverage and the energy used during coating. Consequently, improvements in powder rheology are not an intrinsic property of MgSt but evidence that effective surface modification has been achieved.

Traditional measurements such as bulk and tapped density remain useful during manufacturing operations, including capsule and blister filling [64,65,66,67,68]. However, these parameters provide little information about the interparticulate interactions governing powder dispersion during inhalation. Powder rheometry offers a more relevant assessment because it characterizes dynamic flowability, shear behaviour, cohesion, and aeration properties that are directly related to aerosolization [69,70].

Among rheological descriptors, basic flow energy (BFE), specific energy (SE), and powder shear stress (PSS) characterize powder resistance to flow [71,72], whereas aeration energy (AE) and aeration ratio (AR) reflect particle cohesion and sensitivity to fluidization. Collectively, these parameters allow assessment of whether MgSt coating has effectively modified the powder rather than merely been incorporated into the formulation.

Experimental evidence consistently demonstrates that the rheological benefits of MgSt depend on coating efficiency. Zhou et al. showed that mechanofusion of cohesive lactose with MgSt reduced the specific energy by approximately 50% and decreased powder cohesion from 1.88 to 0.47 kPa, whereas low-shear blending produced essentially no change relative to untreated lactose [47]. Similar observations indicate that MgSt improves flowability only when sufficient process energy is available to disperse MgSt and coat individual particles. Otherwise, MgSt remains in the blend as agglomerates with little functional effect.

The influence of MgSt concentration is equally formulation-dependent. Li et al. observed progressive reductions in BFE following high-shear blending at MgSt concentrations between 0.1 and 0.25%, whereas increasing the concentration to 1% increased BFE again, suggesting that excessive MgSt may produce over-lubrication and deteriorate powder rheology [71]. These findings indicate that coating quality, rather than nominal MgSt concentration, governs rheological performance.

### 6.2. Improving Aerosolization

Improved aerosolization represents the principal objective of MgSt coating, although the underlying mechanisms differ substantially depending on whether micronized APIs or coarse carrier particles are modified.

For cohesive micronized particles, MgSt primarily reduces particle cohesion, thereby facilitating powder fluidization and dispersion during inhalation [73,74]. Begat et al. demonstrated that mechanofusion with 5% MgSt markedly increased the aerosolization performance of carrier-free salbutamol and budesonide formulations, increasing the fine particle fraction (FPF) of salbutamol from 29.18% to 79.42% of the emitted dose [73]. Similar findings were reported independently for mechanofused salbutamol [9].

These studies clearly demonstrate that effective surface modification can substantially improve aerosolization. However, their clinical relevance should be interpreted cautiously. Carrier-free systems containing approximately 95% API are primarily intended for high-dose delivery, whereas most bronchodilators and inhaled corticosteroids are administered in microgram quantities. Consequently, commercial DPI formulations usually employ lactose carriers, which facilitate dose metering and processing while allowing the adhesive interactions governing aerosolization to be tuned.

In carrier-based formulations, MgSt performs a different function. Rather than reducing cohesion alone, it modifies the balance between cohesive and adhesive forces by coating the carrier surface. This surface modification weakens API–carrier adhesion sufficiently to promote drug detachment during inhalation while maintaining blend stability during manufacturing.

Both mechanofusion and high-shear blending have been successfully used to coat lactose carriers. Mechanofusion-treated carriers produced higher FPD than untreated carriers after blending with the API [75]. Likewise, Jetzer et al. demonstrated that only high-shear blending generated a continuous MgSt coating on lactose particles, whereas low-shear blending left MgSt as isolated agglomerates [76]. The resulting reduction in API–carrier and lactose–lactose interactions enhanced drug detachment and increased lung delivery [77,78].

This behaviour has frequently been interpreted within the framework of the active-site theory, according to which MgSt partially masks high-energy adhesion sites on lactose particles [79]. Jetzer et al. further proposed that weakening these interactions promotes formation of API–fine lactose multiplets, which disperse more efficiently than individual API particles and contribute to higher FPD [16,80]. The proposed mechanism is illustrated schematically in Figure 5.

The experimental studies summarized in Table 4 demonstrate that the magnitude of the improvement in aerosolization depends strongly on the coating method, MgSt concentration, and characteristics of the particles being modified.

Although this mechanism is widely cited, direct experimental evidence linking active-site masking with improved aerosolization remains limited. The relative contribution of surface energy reduction, fine-lactose multiplet formation, and changes in electrostatic interactions is still not fully resolved, highlighting an important area for future investigation.

### 6.3. Improving Moisture Stability

Protection of dry powder inhalers (DPIs) from moisture is critical for maintaining their quality and ensuring compliance with critical quality attributes. Moisture adversely affects aerosolization by increasing interparticle cohesion, promoting capillary bridge formation and, in lactose-based systems, accelerating recrystallization of amorphous domains generated during processing. Several approaches can be used to minimize the impact of moisture on DPI formulations, including maintaining controlled environmental conditions during manufacturing, using desiccants in the final product packaging, and employing moisture-barrier blister packaging. The incorporation of MgSt into carrier-based formulations represents another approach to improving the moisture resistance of the powder [81].

MgSt mitigates these effects primarily by forming a hydrophobic surface layer that limits water adsorption and suppresses humidity-induced particle adhesion. Consequently, coated formulations generally retain better dispersibility and higher fine particle delivery after storage under elevated relative humidity.

In co-milled systems, an additional mechanism has been proposed. Besides modifying particle surfaces, MgSt appears to reduce moisture-induced recrystallization of amorphous lactose generated during milling, thereby improving long-term formulation stability [58]. Surface-coated carriers similarly exhibit significantly smaller losses in respirable particle fraction than uncoated lactose after storage at high relative humidity.

Although the protective role of MgSt against moisture is consistently reported, the relative importance of hydrophobic surface modification, inhibition of recrystallization, and changes in interparticle interactions has not yet been systematically quantified. This represents another important knowledge gap that limits direct comparison between different coating technologies and formulation strategies.

## 7. Is There an Optimal Magnesium Stearate Concentration?

The available evidence indicates that no universal magnesium stearate (MgSt) concentration can be recommended for carrier-based DPI formulations. Instead, the optimal level depends on the formulation objective, coating strategy, particle size distribution, specific surface area, and the balance between cohesive and adhesive interactions. Consequently, MgSt concentration should be regarded as a formulation-specific optimization parameter rather than a fixed formulation requirement.

For innovative DPI products, the upper concentration limit is constrained not only by formulation performance but also by toxicological and regulatory considerations. Only approximately 15% of the total dose of MgSt per actuation is estimated to reach the deep lung [78]. However, the FDA lists a maximum potency of 0.13 mg MgSt per unit dose for approved inhalation products [82]. For illustration, if this value is considered in relation to a formulation containing approximately 25 mg of powder per dose, as reported for certain Novartis products [23], it corresponds to roughly 0.52% (*w*/*w*) MgSt. This calculation is based on the specific assumption of a 25 mg total powder dose and represents a conversion of the maximum potency value rather than a general regulatory concentration limit. Nevertheless, it illustrates that formulation optimization is typically performed within a relatively narrow regulatory and safety window.

Experimental studies further demonstrate that increasing MgSt concentration does not necessarily improve formulation performance. For mechanofused salbutamol, aerosolization reached a maximum at 2% (*w*/*w*) MgSt, whereas higher concentrations reduced performance [9]. This observation suggests that once sufficient surface coverage has been achieved, additional MgSt contributes little to coating efficiency and may instead produce thicker deposits or free MgSt agglomerates that adversely affect powder dispersion. Similar concentration-dependent behaviour has also been reported for powder rheology, where excessive MgSt resulted in over-lubrication and deterioration of flow properties [71].

The optimal concentration also depends on the particles being coated. For micronized APIs, relatively high MgSt levels (typically 2–5%) have been investigated because of their large specific surface area and the need to overcome strong cohesive interactions [9]. Begat et al. demonstrated that 5% MgSt substantially improved aerosolization of carrier-free salbutamol and budesonide formulations, producing the greatest increase in fine particle delivery among the excipients evaluated [73]. However, these concentrations cannot be directly translated to carrier-based formulations because the coating target differs fundamentally.

For lactose carriers, considerably lower MgSt concentrations appear sufficient. Complete carrier surface coverage has been reported at approximately 1% MgSt using both mechanofusion and high-shear blending, as confirmed by XPS and ToF-SIMS analyses [29,49]. Because coarse lactose particles possess a substantially lower specific surface area than micronized APIs, effective coating can be achieved with considerably smaller amounts of MgSt. This observation further supports the conclusion that the optimal concentration is governed primarily by the surface area requiring modification rather than by the nominal composition of the formulation.

These findings indicate that MgSt concentration cannot be optimized independently of the coating process. The amount of MgSt required to achieve functional surface modification depends on the efficiency of particle coating, which is itself influenced by process energy, particle morphology, and the extent of surface coverage. Consequently, concentration alone is a poor predictor of formulation performance unless considered together with the coating method.

For generic DPI products, formulation flexibility is additionally constrained by regulatory requirements. The MgSt content is generally matched to that of the reference product to facilitate demonstration of bioequivalence. Although qualitative or quantitative differences may be acceptable, any deviation must be justified by demonstrating that it does not adversely affect critical quality attributes or other aspects of product performance, including patient acceptability [83].

## 8. Conclusions

The available evidence demonstrates that magnesium stearate has evolved beyond its traditional role as a pharmaceutical lubricant and is now used as a functional surface modifier or force control agent in carrier-based dry powder inhalers. MgSt can influence powder rheology, interparticulate interactions, aerosolization efficiency, and resistance to humidity. However, these effects are not intrinsic to MgSt itself but depend on the interplay between material properties, coating process, and formulation design.

A consistent finding across the reviewed studies is that coating efficiency depends strongly on process energy and the characteristics of the particles being modified. High-energy techniques, particularly mechanofusion, are capable of coating highly cohesive micronized particles that cannot be effectively modified by conventional blending, whereas high-shear blending may provide sufficient energy for coarse lactose carriers. Direct comparison between coating technologies remains difficult because studies differ in MgSt grade, carrier and API properties, particle-size distribution, processing conditions, and methods used to assess coating efficiency. Thus, no single coating technique can be considered universally optimal.

MgSt generally improves aerosolization by modifying cohesive and adhesive interactions, but the magnitude of this effect is formulation-specific. Fine particle delivery depends not only on MgSt concentration but also on particle size and surface area, carrier properties, coating efficiency, and inhaler design. Increasing MgSt concentration does not necessarily improve performance; once sufficient surface modification has been achieved, additional MgSt may provide little benefit and, in some systems, may promote overlubrication or the formation of agglomerates that compromise powder flow and aerosolization. Consequently, MgSt concentration should be optimized together with the coating process and the properties of the particles being modified.

Important knowledge gaps remain. Most studies infer coating efficiency indirectly from aerodynamic performance, whereas direct characterization of surface coverage using techniques such as XPS or ToF-SIMS remains relatively uncommon. The quantitative relationships between coating extent, surface properties, powder rheology, moisture resistance, and aerosolization are therefore still poorly defined. Similarly, the relative contributions of reduced surface energy, active-site masking, fine-lactose multiplet formation, electrostatic interactions, and moisture protection remain incompletely understood. Methodological differences between studies further limit mechanistic interpretation and direct comparison of coating strategies.

Future research should therefore move beyond demonstrating that MgSt improves DPI performance toward establishing why, how, and under which conditions these improvements occur. Greater methodological standardization, direct characterization of surface modification, and systematic integration of material attributes, process conditions, coating characteristics, and aerodynamic performance are needed. Particular attention should be given to the relationship between process energy, particle size, surface area, and cohesion, as these factors determine both coating efficiency and the amount of MgSt required for effective surface modification. Developing predictive approaches that integrate these variables could support a transition from empirical optimization toward rational formulation design. MgSt should therefore not be regarded as a universally beneficial additive, but as a particle-engineering excipient whose functionality must be tailored to the specific API–carrier–process–device system.

## Figures and Tables

**Figure 1:**
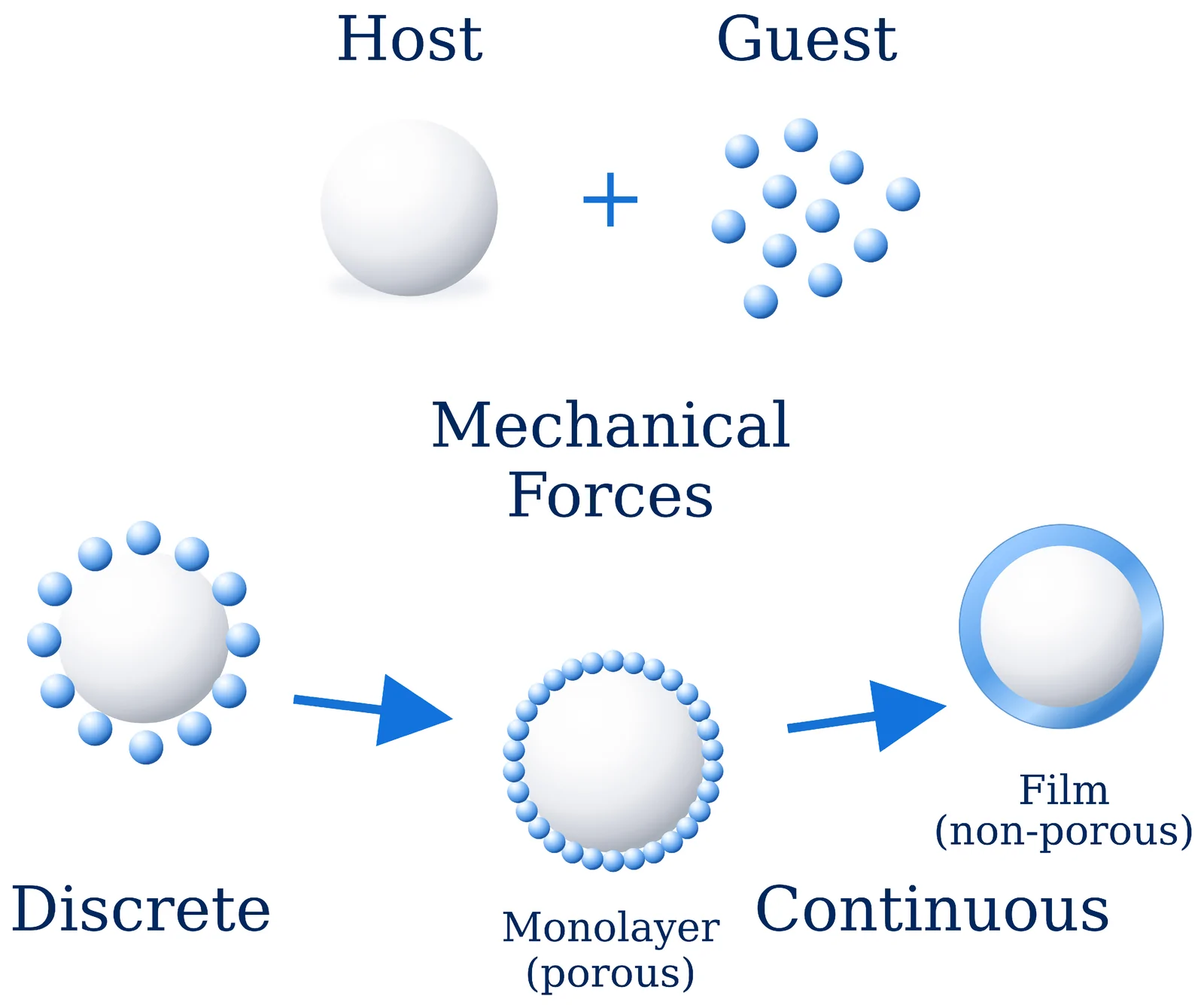
Schematic of dry particle coating.

**Figure 2:**
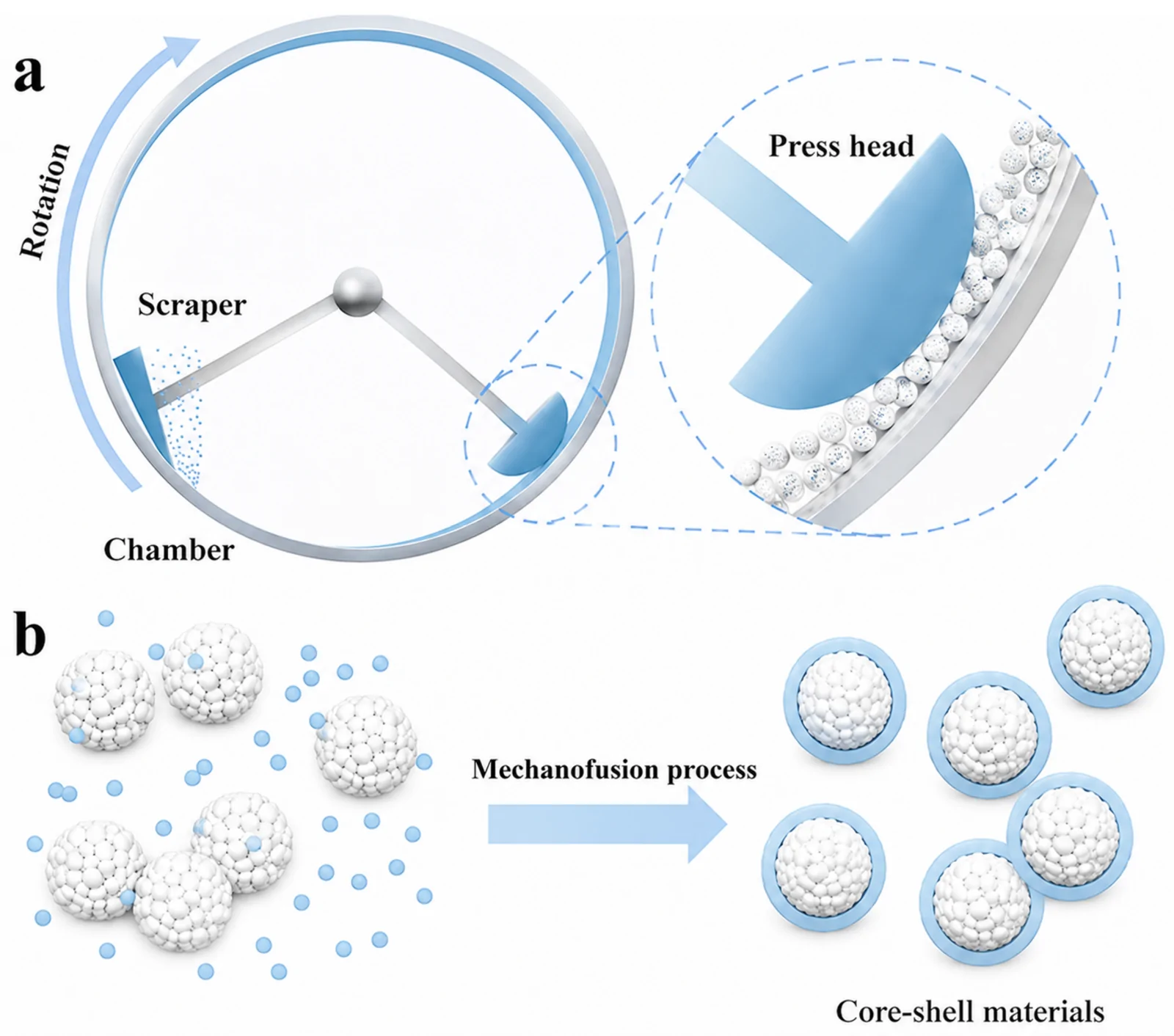
Schematic illustration of the mechanofusion process (**a**) and mechanofused materials (**b**) ([44], modified).

**Figure 3:**
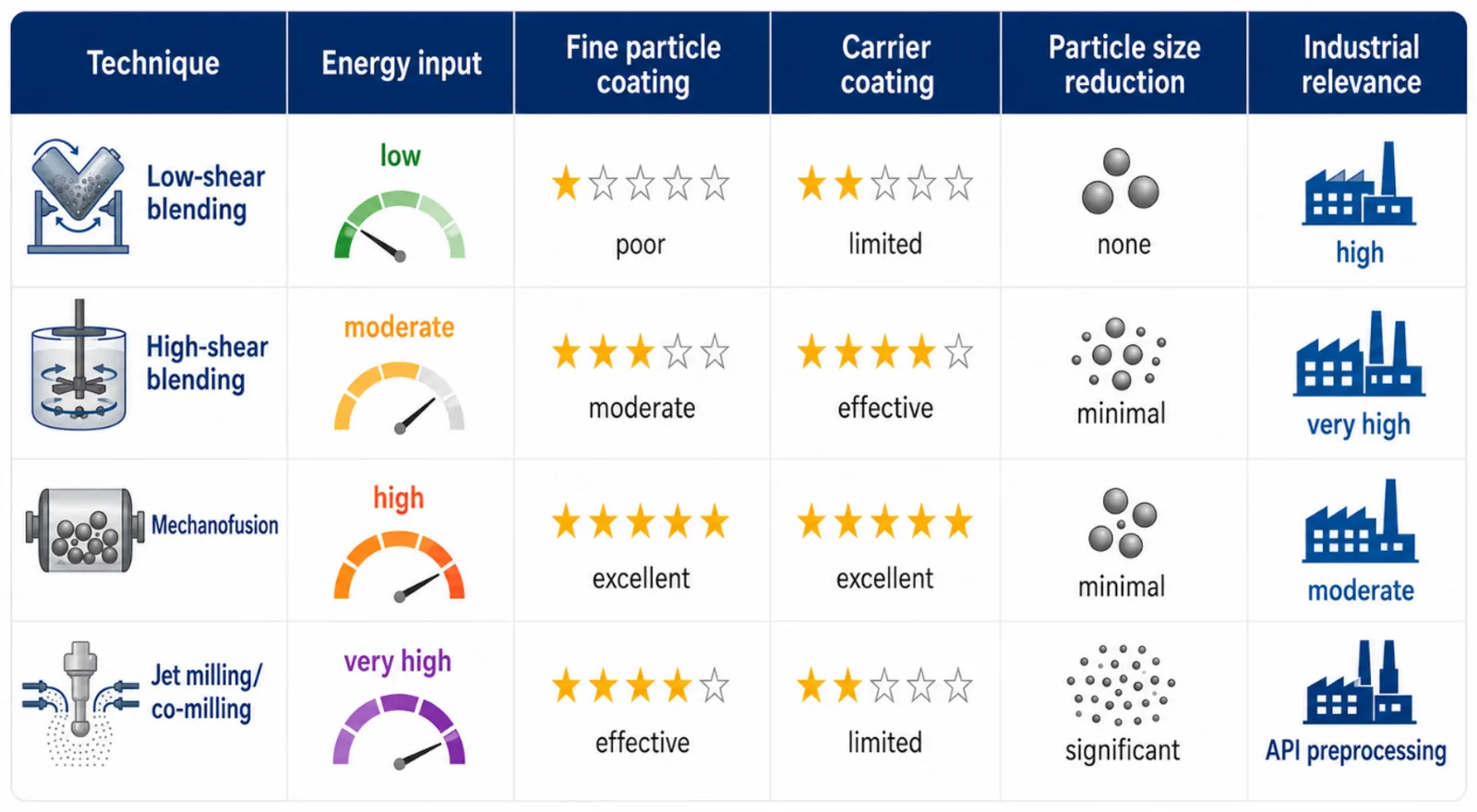
Comparison of the techniques used for coating carrier particles with magnesium stearate.

**Figure 4:**
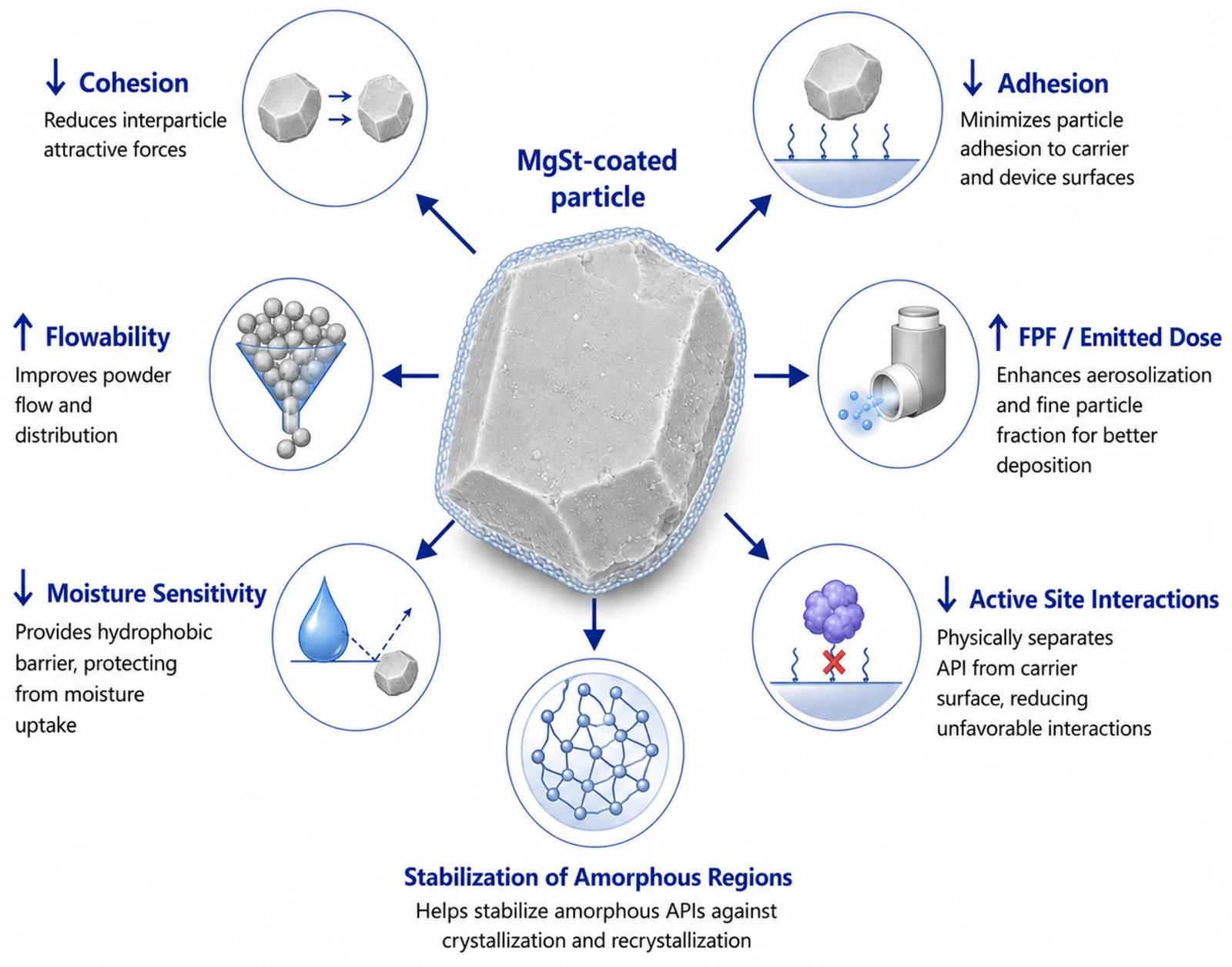
Multifunctional role of magnesium stearate in carrier-based DPI formulations.

**Figure 5:**
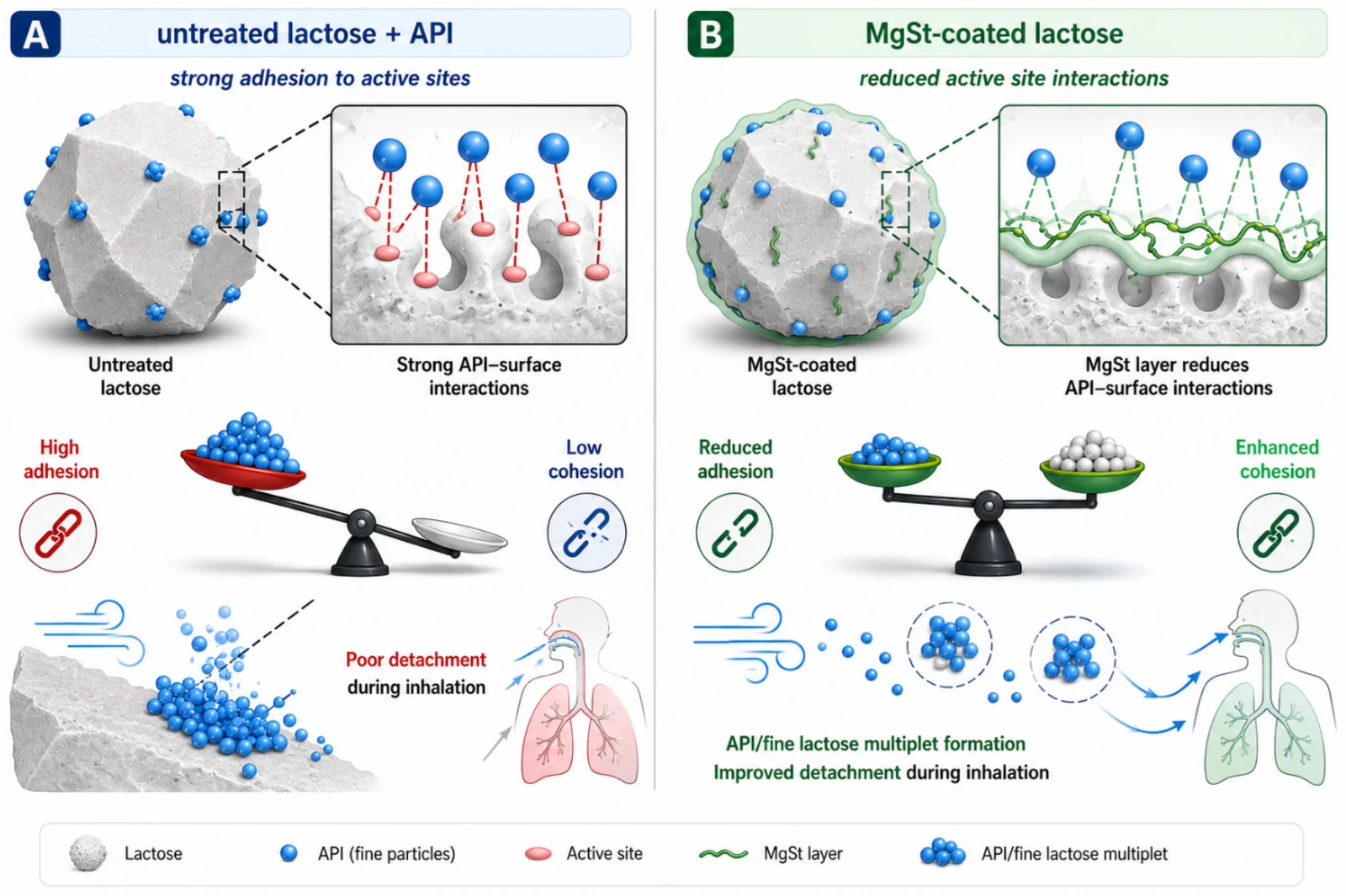
Schematic of cohesive–adhesive balance before and after MgSt coating.

**Table 1:** Comparison of vegetable- vs. bovine-derived magnesium stearate [31].

Property	Vegetable-Derived MgSt	Bovine-Derived MgSt
Source	Plant oils (e.g., palm, coconut)	Animal fats (e.g., bovine tallow)
Regulatory/acceptability	Preferred (non-animal, lower contamination risk)	Limited use due to BSE/TSE * concerns
Crystallinity	Higher crystallinity	More amorphous character
Particle size	Typically smaller, more uniform	Larger, more variable
Specific surface area	Higher (in some studies comparable but functionally more effective)	Lower or similar but less effective in lubrication
Particle morphology	Plate-like/rounded particles	Irregular, needle-like particles
Moisture interaction	More structured hydration (crystalline lattice)	Greater amorphous water association
Surface properties	Lower surface tension, higher zeta potential (better dispersion)	Higher surface tension, lower zeta potential
Fatty acid composition	Higher purity (C16–C18 dominant ~99%)	More heterogeneous (broader fatty acid profile)
Flow properties	Generally similar (poor flow typical for both)	Similar flow characteristics
Lubrication efficiency	Generally higher (lower ejection force, better film formation)	Lower or more variable lubrication efficiency
Functional performance (tableting/DPI relevance)	Better coating, reduced adhesion, improved dispersion	Less efficient surface modification, stronger particle interactions
Overall performance consistency	More reproducible and predictable	Greater batch-to-batch variability

* BSE: Bovine Spongiform Encephalopathy; TSE: Transmissible Spongiform Encephalopathies.

**Table 2:** EMA- and/or FDA-approved lactose-based DPI products without magnesium stearate as of September 2026.

Active Ingredient	Excipients	Brand Name	Manufacturer	Marketing Authorisation
Salmeterol xinafoate	Lactose	Serevent Diskus	GlaxoSmithKline	1997
Fluticasone propionate + Salmeterol	Lactose	Advair Diskus	GlaxoSmithKline	2000
Tiotropium bromide	Lactose	Spiriva Handihaler	Boehringer Ingelheim	2004
Indacaterol maleate	Lactose	Onbrez Breezhaler	Novartis	2009
Aclidinium bromide	Lactose	Tudorza Pressair	Covis Pharma	2012
Fluticasone furoate	Lactose	Arnuity Ellipta	GlaxoSmithKline	2014
Albuterol sulfate	Lactose	ProAir RespiClick	Teva	2015
Aclidinium bromide, Formoterol fumarate	Lactose	Duaklir Pressair	Covis Pharma	2019

**Table 3:** FDA- and EMA-approved lactose-based DPI products containing magnesium stearate as of September 2026.

Active Ingredient	Excipients	Brand Name	Manufacturer	Marketing Authorisation
Glycopyrronium bromide	Lactose MgSt	Seebri Breezhaler	Novartis	2012
Glycopyrronium bromide, Indacaterol maleate	Lactose MgSt	Ultibro Breezhaler	Novartis	2013
Fluticasone furoate, Vilanterol	Lactose MgSt	Relvar Ellipta	GlaxoSmithKline	2013
Umeclidinium	Lactose MgSt	Incruse Ellipta	GlaxoSmithKline	2014
Umeclidinium bromide, vilanterol trifenatate	Lactose MgSt	Anoro Ellipta	GlaxoSmithKline	2014
Fluticasone furoate, umeclidinium bromide, vilanterol trifenatate	Lactose MgSt	Trelegy Ellipta	GlaxoSmithKline	2017
Glycopyrronium bromide, Indacaterol acetate, Mometasone Furoate	Lactose MgSt	Enerzair Breezhaler	Novartis	2020

**Table 4:** Selected studies evaluating the effect of MgSt surface modification on DPI aerosolization performance.

API	Carrier	Process	MgSt (% *w*/*w*)	Key Outcome	Reference
Salbutamol; Budesonide	None	Mechanofusion	5	FPD ↑; salbutamol: 29.2 → 79.4% ED ***	[73]
Salbutamol	None	Mechanofusion	0.5–10	Best at 2%; 71.4 → 81.0% ED	[9]
Ipratropium	InhaLac^®^ 230	Mechanofusion/HSB *	2	FPF ↑ 128%/120%	[46]
Fluticasone; Salmeterol	Respitose^®^ ML001	HSB/LSB **	0.1–10	HSB improved FPF; best ≈ 0.5%	[76]
Fluticasone	Respitose^®^ SV003; Lactohale^®^ 206/100	HSB/LSB	0.1–1	Best ≈ 0.25–0.5%; higher FPF with LSB	[71]

* HSB: high shear blending; ** LSB: low shear blending; *** ED: emitted dose.

## Data Availability

No new data were created or analyzed in this study. Data sharing is not applicable to this article.
